# Distribution of soil viruses across China and their potential role in phosphorous metabolism

**DOI:** 10.1186/s40793-022-00401-9

**Published:** 2022-02-07

**Authors:** Li-Li Han, Dan-Ting Yu, Li Bi, Shuai Du, Cynthia Silveira, Ana Georgina Cobián Güemes, Li-Mei Zhang, Ji-Zheng He, Forest Rohwer

**Affiliations:** 1 State Key Laboratory of Urban and Regional Ecology, Research Center for Eco-Environmental Sciences, Chinese Academy of Sciences, Beijing, 100085 China; 2 University of the Chinese Academy of Sciences, Beijing, 100049 China; 3 Department of Biology, San Diego State University, San Diego, CA 92182 USA; 4 Key Laboratory for Subtropical Mountain Ecology of the Ministry of Science and Technology and Fujian Province, School of Geographical Sciences, Fujian Normal University, Fuzhou, 350007 Fujian China; 5 College of Resources and Environment, Huazhong Agricultural University, Wuhan, 430070 China; 6 Viral Information Institute at San Diego State University, San Diego, CA 92182 USA; 7 Department of Biology, University of Miami, Coral Gables, FL 33146 USA

**Keywords:** Virus, Virome, Geographic location, PhoH, P metabolism, Nucleotide synthesis

## Abstract

**Background:**

Viruses are the most abundant biological entities on the planet and drive biogeochemical cycling on a global scale. Our understanding of biogeography of soil viruses and their ecological functions lags significantly behind that of Bacteria and Fungi. Here, a viromic approach was used to investigate the distribution and ecological functions of viruses from 19 soils across China.

**Results:**

Soil viral community were clustered more significantly by geographical location than type of soil (agricultural and natural). Three clusters of viral communities were identified from North, Southeast and Southwest regions; these clusters differentiated using taxonomic composition and were mainly driven by geographic location and climate factors. A total of 972 viral populations (vOTUs) were detected spanning 23 viral families from the 19 viromes. Phylogenetic analyses of the *phoH* gene showed a remarkable diversity and the distribution of viral *phoH* genes was more dependent on the environment. Notably, five proteins involved in phosphorus (P) metabolism-related nucleotide synthesis functions, including dUTPase, MazG, PhoH, Thymidylate synthase complementing protein (Thy1), and Ribonucleoside reductase (RNR), were mainly identified in agricultural soils.

**Conclusions:**

The present work revealed that soil viral communities were distributed across China according to geographical location and climate factors. In addition, P metabolism genes encoded by these viruses probably drive the synthesis of nucleotides for their own genomes inside bacterial hosts, thereby affecting P cycling in the soil ecosystems.

**Supplementary Information:**

The online version contains supplementary material available at 10.1186/s40793-022-00401-9.

## Introduction

Viruses are the most abundant and diverse biological entities form and are major contributors to ecosystem functioning across all habitats [1]. Previous studies showed that viruses shape marine ecosystems by controlling the abundance and genomic diversity of their hosts through cell lysis [2–4] or lysogeny [5], and horizontal gene transfer [6–9]. Compared to around 1.01 × 10^29^ virus-like particles (VLPs) in marine environments, approximately 4.88 × 10^30^ VLPs were estimated to reside in global soils, accounting for 10% of the global viral abundance (4.80 × 10^31^) [1]. The potential roles of soil viruses in terrestrial ecosystem processes include impacting microbial mortality, biogeochemical cycling of soil elements, and food web dynamics [10]. Although soil viromes only contribute less than 1% of publicly available viral metagenomes [1], an increasing number of studies of viromes have focused on various soils, such as desert soil [11, 12], glacier soil [13], thawing permafrost soil [14], mangrove soil [9], mud volcanic soil [15], and Antarctic soil [16]. These studies revealed different patterns of soil viral community structure and largely uncharacterized viral assemblages. However, only a few studies have offered insight into how environmental factors influence viral communities. Soil pH was the main environmental driver of the viral community structure in agricultural soils [17]. Except soil pH, calcium content and site altitude were the main drivers of the Antarctic viral community structure [16].

In Chinese agricultural ecosystems, phosphorus (P) is an important biologically limiting nutrient that must be heavily supplemented for improving crop production [18]. Though lots of chemical P fertilizers have been applied to agricultural land, the P availability is still very low due to P slow diffusion and high fixation in soils [19]. Previous studies showed that P content in the marine ecosystem could affect the proportion of P allocated from hosts to viruses, as viruses have a higher proportion of P (C/N/P ≈ 20/6/1) [20] than Bacteria (69/16/1) [21, 22]. We considered the possibility that viruses in the soil ecosystem may also accelerate the uptake of soil P to synthesize their own genomes when P fertilizers were supplemented by the host cell. Thus, viral infection could cause the P present in the host bacteria to be disproportionately incorporated into the new phage particles, further resulting in P removal from soil biotic cycling and affecting plant and microbial P acquisition strategies [23]. However, it is not clear how viruses manipulate this process and whether this process is related to the P concentration or P fertilizer input in the soils.

Increasing evidence has shown that a certain number of putative auxiliary metabolic genes (AMGs) encoded by viruses are expressed during the infection cycle, and that AMG products reprogram host cell metabolism with direct impacts on biogeochemistry cycling [7, 24, 25]. In the genomes of globally abundant ocean viruses, more than two hundred viral-encoded AMGs have been identified [8], including carbon, nitrogen, sulfur, and P cycle related genes. Some viral AMGs, such as *trzN* [26], *phoH* [11], RNR [11], *spoIIIE* [27], carbon cycling related genes (CAZymes [9, 17], central C metabolism genes [14]), and oxidative phosphorylation related genes etc. [28] have been identified in soil ecosystems. Among them, the *phoH* gene encodes an ATP binding protein with undetermined function [29] and is presumed to belong to the Pho regulon and to regulate P uptake and metabolism under low-phosphate conditions [30]. It is known to be induced under phosphate stress in *E. coli*, while its expression is not upregulated during P starvation in marine cyanobacteria [31–33]. Despite the *phoH* gene is found widely distributed among both eubacteria and archaea [34, 35], our knowledge of their functions and potential mechanisms is still a mystery.

In this study, we aimed to investigate the distribution of viral communities and functions from 19 soil samples across China, and determine the main factors driving viral distribution and function. Furthermore, we explored whether the *phoH* gene and its homologs may play important roles in P cycling in soil ecosystems.

## Materials and methods

### Soil sampling and physicochemical properties

Between August 2015 and August 2016, a total of 19 soil samples were collected from ten provinces across China; these samples included ten agricultural soil samples and nine natural soil samples (Additional file 1: Fig. S1 and Additional file 2: Table S1). The agricultural soil samples, from five maize fields and five paddy fields, were located in seven provinces. The natural soil samples were also located in seven provinces and included forest, grassland, wetland, coastal, glacier, and mud volcanic soils (Additional file 2: Table S1). To study viral diversity and function in these soils, approximately 5 kg of each sample was collected and transported at 4°C back to the laboratory. At each site, a soil sample was collected from each of three separated 10 m × 10 m plots by pooling five upper 20-cm soil cores randomly taken from every plot. The three samples from each site were pooled and then processed as follows: 1 kg of soil was sieved to 1 mm for virus extraction, and 500 g of each soil sample was sieved to 2 mm and then stored at 4°C for physicochemical analyses.

A pH meter (Professional Meter PP-20, Sartorius, Germany) was used to measure soil pH and electrical conductivity (EC) at a ratio of 1:2.5 and 1:5 (soil to water, w/w), respectively. Organic matter (OM) was determined using the K_2_Cr_2_O_7_ oxidation method. Total nitrogen (TN) was measured using a Vario EL III analyzer (Elementar Analysensysteme GmbH, Hanau, Germany). Available P was determined using the Olsen method [36]. Available potassium (AK) was extracted with 0.5 M ammonium acetate and quantified using an atomic absorption spectrophotometer (ZEEnit700P, Analytik Jena AG, Jena, Germany). Mean annual temperature (MAT) and mean annual precipitation (MAP) data were from WorldClim Version2.

### Virus extraction and purification

Viruses were extracted from the soil samples according to the method of Williamson et al. [37]. Briefly, 500 g of soil per sample was suspended in 1.5 L of glycine buffer (250 mM; pH = 8.5), shaken for 30 min, and centrifuged at 4000 g for 10 min at 4°C to precipitate soil particles. The supernatant was filtered sequentially through 1-mm, 0.45-µm, 0.20-µm tangential flow filters (GE Healthcare Life Sciences, Pittsburgh, PA, USA), and concentrated the filter liquid to less than 100 ml by 30-kDa tangential flow filters (GE Healthcare Life Sciences, Pittsburgh, PA, USA). The viruses in the filtrate were further concentrated using 30-kDa centrifugal ultrafiltration tubes (Merck Millipore Ltd., Tullagreen, Ireland) until the final sample volume was less than 1 ml. Finally, viral concentrates were treated with DNaseI (10 units DNaseI/100 μl) and incubated at 37°C for 1 h to remove free, non-encapsulated DNA. The presence of free and contaminating bacterial DNA was checked by PCR amplification of the 16S rRNA gene with primers 27F/1492R [38].

### Viral DNA extraction and high-throughput sequencing

The Power Viral Environmental RNA/DNA Isolation kit (MO BIO Laboratories, Carlsbad, CA, USA) was used to extract total DNA. The REPLI-g Mini Kit (for multiple displacement amplification (MDA)) (Qiagen, Hilden, Germany) using Phi29 polymerase was applied to transfer ssDNA to dsDNA and obtain the concentration and quantity needed for high-throughput sequencing. For each sample, more than 1 ng of DNA was fragmented to approximately 400 bp and used as a template to create a metagenome library, which was constructed according to the TruSeq™ DNA Sample Prep Kit (Illumina, San Diego, CA, USA) protocol. The libraries were loaded onto flow cell channels for sequencing using an Illumina HiSeq2500 at Shanghai Majorbio Bio-pharm Biotechnology Co., Ltd. (Shanghai, China) to generate 300-bp paired-end reads.

### Analysis of viromes

#### Data sets and assembly

The original raw reads of the 19 samples obtained from the Illumina HiSeq2500 were cleaned using Fastp software [39] for quality filtering and subsample the raw data. Firstly, adapter bases or poly [ATCG] bases (minlength = 10) in the 5′ or 3′ reads were removed. Secondly, those reads were deleted that meet any one of the following conditions: the number of N bases in the sequence exceeds 5 bp, the average sequence quality value QV < 20, sequence length < 18 bp, or sequence complexity < 30%. After quality control, each sample was independently assembled using metaSpades with default parameters [40], and contigs shorter than 10 kb were eliminated according to Minimum Information about an Uncultivated Virus Genome (MIUViG) [41]. A combination of VirSorter [42], VIBRANT [43] and DeepVirFinder [44] were used to detect viral contigs from each assembly. Based on the Discovery Environment 2.0 (https://de.cyverse.org), Virsorter was run in decontamination mode, and only categories 1, 2, 4 and 5 (higher confidence predictions) were retained, and combined phages in VIBRANT were considered viral. DeepVirFinder run according to its python script (https://github.com/jessieren/DeepVirFinder), and contigs with scores > 0.9 and *p* < 0.05 were considered viral [45]. All resulting viral contigs were combined and clustered at 98% identity with cd-hit-est software [46], resulting in 972 non-redundant genome fragments to create a viral Operational Taxonomic Units (vOTUs) database. Frap [1] was used to map quality-filtered reads from each sample to the vOTU database at 90% identity, with the genome size normalization option, to obtain the normalized vOTU table. Normalization was done by dividing the number of reads aligned database by the number of reads in the virome, then multiplying this by the mean genome length divided by the length of each viral contig. The number of viral reads was calculated by reads aligning to these vOTUs.

#### Viral taxonomy clusters and potential impact factors

An unsupervised random forest analysis was used to cluster the samples based on the normalized vOTU table and identify which environmental and/or geographical factors influenced viral community composition using the "randomForest" and "rfPermute" packages on the R platform [47]. Non-metric multi-dimensional scaling (NMDS) was used to analyze the random forest proximity matrix, to cluster the samples based on Ward distances, and to identify the subset of variables of importance for the random forest clustering. The effect of environmental factors and geographical coordinates on this dataset was tested using a supervised random forest permutational-based variable importance measures to identify the significant predictors of viral community composition.

#### Taxonomy annotation and comparison

Clean reads were classified using Kraken2 against the NCBI viral reference sequences (minikraken2_v1_8GB_201904) to identify viral reads [48]. The abundance of viral reads was computed by Bracken [49], which uses the taxonomy labels assigned by Kraken2 to estimate the number of reads present in each sample. UpSet analysis was further performed to visualize the interactive viral families among clusters by the "UpSetR" on the R platform [50].

#### Putative viral AMGs detection

DRAM-v [51] was used to identify putative AMGs in 972 vOTUs, and only results with viral_bitScore > 60 and viral_E-value < 1E−5 were retained. The contigs containing genes related to the P metabolism module were selected from the DRAM-v output, including genes encoding the predicted proteins dUTPase [52], MazG, PhoH, RNR, and Thy1 [53, 54]. Genomic maps of contigs encoding the *phoH* gene were generated with Easyfig [55].

#### Phylogenetic analysis of the *phoH* gene

Phylogenetic trees of the *phoH* gene amino acid sequences were reconstructed using MEGA-X software [56]. A total of 102 representative *phoH* gene amino acid sequences from viruses were collected (Additional file 2: Table S2), including 25 reference sequences from cultured phages, 15 from paddy water [57], 8 from sea water [7, 30], 9 form permafrost soil samples [58], 12 from other soil metagenomes [59], 25 reference sequences of soil viromes obtained from our previous work [17], and 8 sequences in this study. All selected amino acid sequences were aligned by ClustalW, and the gaps and ambiguously aligned positions were deleted. After alignment, a phylogenetic tree was constructed using the Jones–Taylor–Thornton (JTT) model and the maximum likelihood method, and support for tree structure was obtained using 1000 bootstraps. The output was visualized by Evolview v2 [60].

### Data availability

Virome read data are available in the NCBI Short Read Archive (SRA) under BioProject ID PRJNA579576.

## Results

### Viral community structure

Soil samples from 10 provinces across China were used to generate 19 soil viromes, including 10 agricultural soil viromes and 9 natural soil viromes (Additional file 1: Fig. S1). A total of 186,503,518 reads (range: 4,782,132 to 15,945,343 per sample) passed quality control. Among them, 433,669 reads were identified as viral by Kraken 2, and most were still unclassified (Additional file 2: Table S3). A total of 972 de-replicated viral contigs (> 10 kb) were assembled and reserved for further analysis according to MIUViG [41]; the longest contig was 98,359 bp and the average contig length was 19,760 bp.

**Fig. 1:**
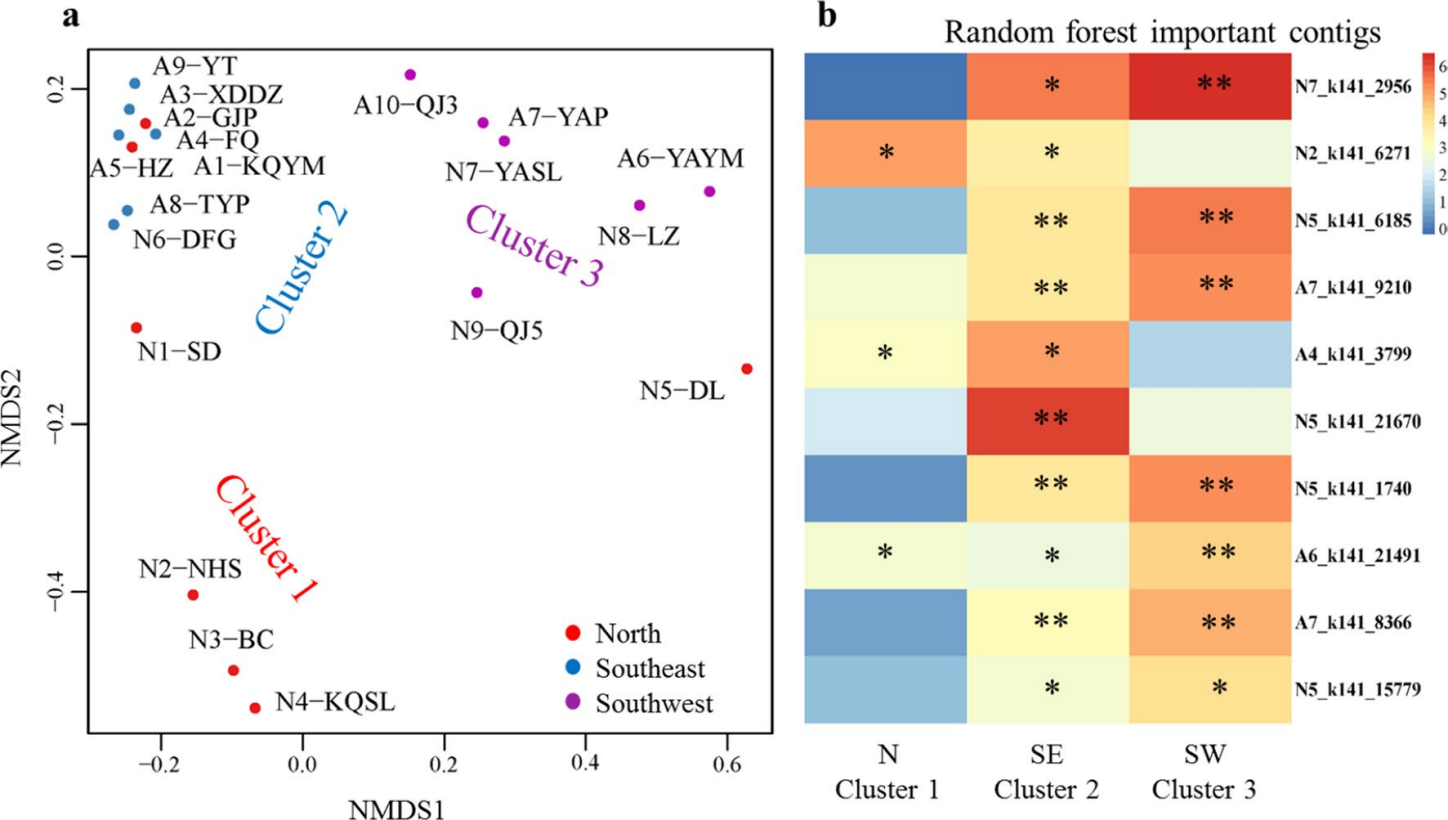
Viral community structure. **a** Non-metric multidimensional scaling (NMDS) of viral community composition in 19 soil viromes obtained from an unsupervised random forest analysis followed by clustering using Ward distances (OOB estimate of error rate = 21.05%). Symbols are color-coded by site (red: North sites; blue: Southeast sites; purple: Southwest sites). **b** The heat map of contigs differentiating the geographical clusters in a random forest analysis supervised by geographical location and that there is a color gradient which represents the importance. *P* < 0.01, **; *P* < 0.05, *

To identify similarities between soils, soil viromes were clustered using an unsupervised random forest analysis based on vOTU table (Additional file 2: Table S4). Three clusters of samples were identified (21.05% OOB (out-of-bag) estimate of error rate), and were related to the geographical distribution of the soil samples (Fig. 1a and Additional file 1: Fig. S1). Cluster 1 included four of seven North China samples, Cluster 2 included all six Southeast China samples, and Cluster 3 included all six samples from the Southwest of China (Fig. 1a and Additional file 1: Fig. S1). The top ten contigs with highest importance in differentiating clusters are shown in Fig. 1b and Additional file 1: Fig. S2.

To analyze and compare the viral community composition with respect to environmental factors, soil physical and chemical properties including pH, EC, OM, TN, AP, and AK, climate factors (MAT, Mean annual temperature, and MAP, mean annual precipitation (Additional file 2: Table S1)), and geographical coordinates were tested as potential predictors of viral frequencies in the vOTU table. The results indicated that only MAP, MAT, longitude, and latitude explained 12.78%, 8.2%, 21.18% and 22.08% of the variation in viral community composition, respectively. Soil physical and chemical properties didn’t show any relationship with viral community composition.

**Fig. 2:**
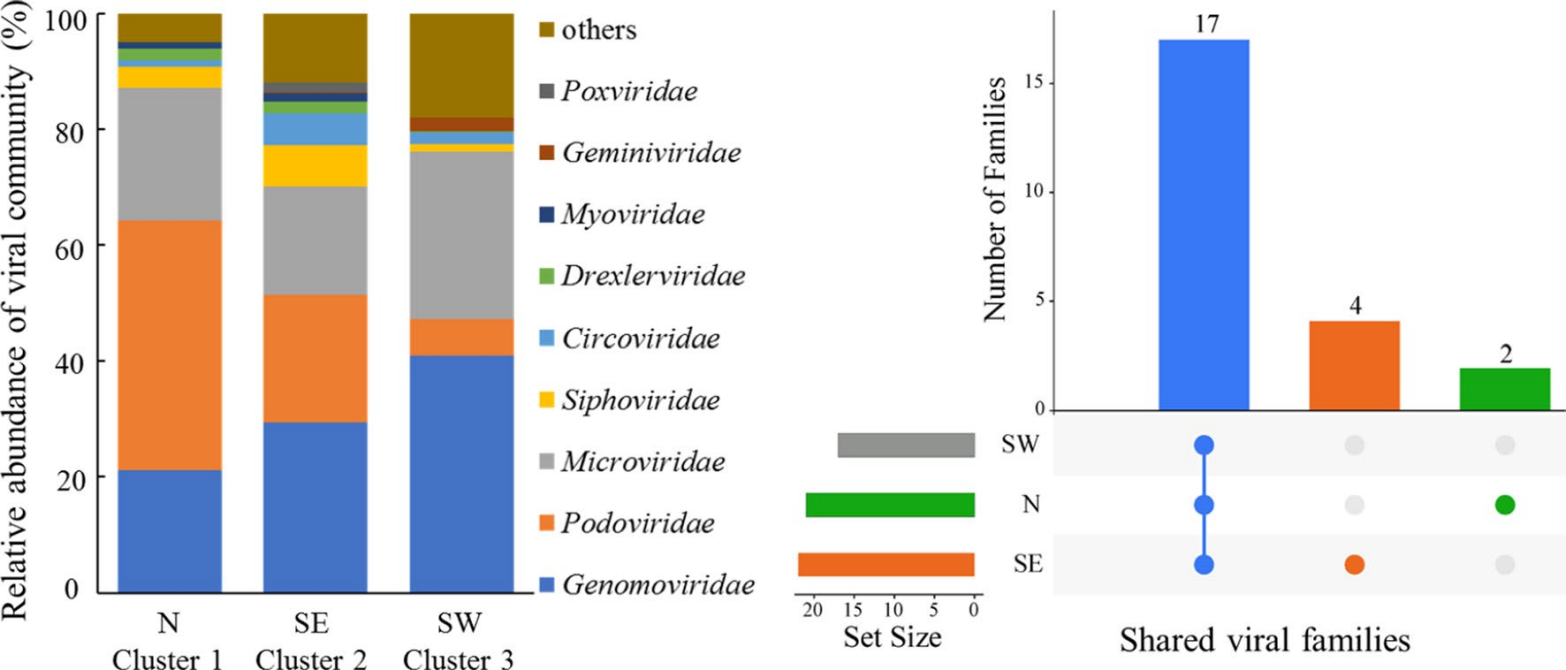
Viral taxonomy. **a** Relative abundances of the viral families among three NMDS clusters (top 10 are shown), and MDA was used in this study. **b** Shared viral families among three clusters displayed by UpSet analysis

A total of 23 viral families (Fig. 2a, b and Additional file 2: Table S5) were identified from the 19 viromes by Kraken 2, including 15 families belonging to dsDNA viruses and eight families of ssDNA viruses. For ssDNA viruses, the *Microviridae*, *Genomoviridae* and *Circoviridae* families were widespread in all clusters. For dsDNA viruses, the *Caudovirales* (tailed viruses that infect Bacteria and Archaea) including *Myoviridae*, *Siphoviridae*, and *Podoviridae* were widespread in all clusters. Meanwhile, few numbers of giant viruses (*Mimiviridae* and *Pandoraviridae*) were distributed in all three clusters. In addition to these shared viruses, there were some specific viruses in Cluster 1 (North) and Cluster 2 (Southeast) (Fig. 2b). Such as, *Anelloviridae* and *Hepadnaviridae* were mainly present in Cluster 1 (North), and *Bacilladnaviridae*, *Demerecviridae*, *Inoviridae*, and *Marseilleviridae* only existed in Cluster 2 (Southeast) (Fig. 2b).

### P metabolism module

**Fig. 3:**
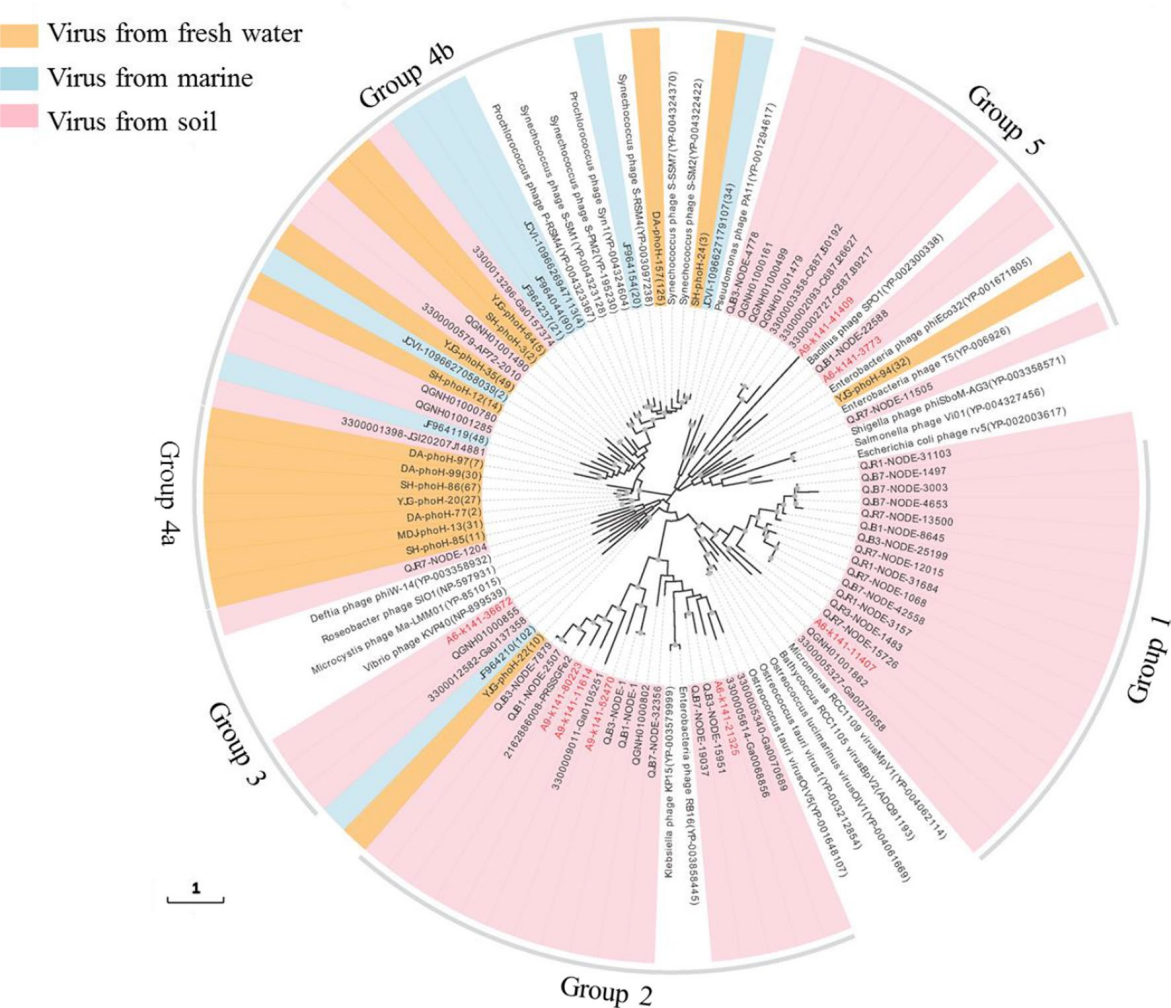
Phylogenetic tree of *phoH* gene sequences. Only representative sequences are displayed in the tree, and the number represents the number of original sequences. Sequences from fresh water, marine and soil are colored in lightyellow, blue and pink respectively. The red font in the figure represents the data from this study. The tree was bootstrapped with 1000 sub-replicates, and bootstrap scores > 50% are flagged with circles

A phylogenetic tree of the *phoH* gene was built with 102 viral amino acid sequences from this study and others (Fig. 3). 15 representatives were collected from fresh water [57], eight representatives from sea water [7, 30], 25 reference sequences from cultured phages, and 54 *phoH* amino acid sequences from soil metagenomes [17, 58, 59] including eight representatives from agricultural maize fields in this study. All of the eight *phoH* amino acid sequences obtained from viromes of agricultural soils. Overall, the phylogenetic tree could be mainly divided into five groups. Group 1, 2, and 3 mainly contained viruses from soil samples, while group 4 and 5 contained viruses from different environments. Six *phoH* gene sequences in this study were grouped into Group 1, 2, and 3 with other global soil samples, and two were clustered into Group 5 with fresh water and other soil samples.

**Fig. 4:**
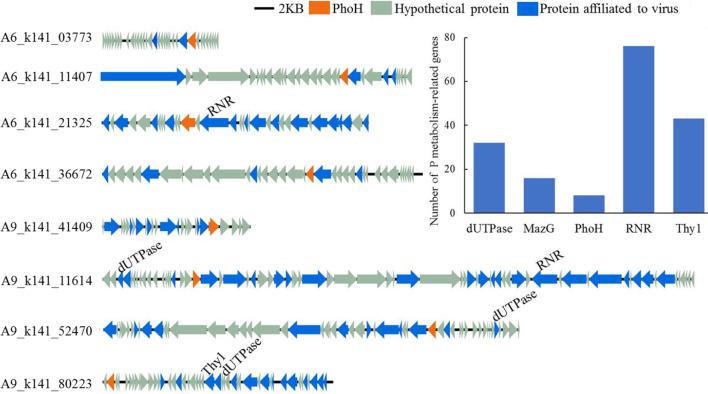
Eight dsDNA viral contigs carrying AMGs predicted to be involved in phosphate metabolism. Arrows indicate ORFs and arrow color indicates predicted function. A histogram showing the number of genes in the Phosphate metabolism module, and the ordinate represents the number of viral ORFs

Putative AMGs were identified using DRAM-v [51]. Genes functionally related to *phoH* were further analyzed, which include five P metabolism-related nucleotide synthesis functions involving dUTPase, MazG, PhoH, Thy1, and RNR. A total of 175 viral ORFs belonging to the five P metabolism proteins were identified (Fig. 4), and they were mainly from agricultural soils (158 of 175 ORFs). Eight representative contigs (> 10 kb, all from maize fields) containing the *phoH* genes belonged to dsDNA viruses, and some accompanied genes encoding dUTPase, Thy1, or RNR (Fig. 4). In addition to these P metabolism proteins, these contigs encoded mostly hypothetical proteins.

## Discussion

Viruses play a vital role in the distribution of organisms and their contributions to global biogeochemical cycles [23]. However, our understanding of soil viruses, and the factors driving their distribution, lags far behind that of marine viruses. Further, the ways in which viruses participate in the biogeochemical cycling of soil elements have not been extensively investigated. This study provides evidence that geographic location and climate factors are key drivers of viral distribution in soils. Furthermore, the higher abundance of viral-encoded P metabolism genes in agricultural soils indicates that viruses have the potential roles of P cycling in these soil ecosystems.

### The taxonomic distribution of soil viruses

The order *Caudovirales*, including *Siphoviridae*, *Myoviridae* and *Podoviridae*, was dominant in all of our soil samples, in agreement with previous studies [9, 11, 12, 16, 61]. In the Antarctic soil, *Podoviridae* presented at similar levels in all samples, whereas the abundances of *Myoviridae* and *Siphoviridae* were inversely correlated, as they may have direct competition for hosts in the same niche, and Siphoviruses are always present at higher abundances in neutral to alkaline pH soils [16]. However, our study showed different trends, with *Myoviridae* occurring in all samples with low abundance, whereas *Siphoviridae* and *Podoviridae* were mainly present in more acidic soils (Additional file 2: Table S5). More data is needed to find patterns, especially since so many viruses in the viromic data could not be classified and we used MDA.

Moreover, our soil viromes revealed diverse ssDNA viruses belonging to the *Microviridae*, *Circoviridae*, and *Genomoviridae*, (Fig. 2a). The broad presence of ssDNA viruses is likely due to the bias of MDA, which preferentially amplifies genomes of ssDNA viruses and thus leads to a quantitative bias [62–64]. Therefore, both ssDNA and dsDNA viruses were reported in a qualitative rather than quantitative way in this study. Meanwhile, the use of MDA leads to many short sequences. In this study, contigs less than 10 kb were ignored to avoid a misunderstanding of the soil virome. However, discovery of unknown function or partial viral genomes is still an important work.

### Geographic location drives viral community composition and function

Viral community composition has been associated with a variety of environmental factors, such as host community composition, pH, soil depth and moisture, calcium content and site altitude [14, 16, 23, 58, 65]. According to the unsupervised random forests analysis, the viral communities and functions from 19 soil samples across China grouped into 3 clusters, which corresponded to geographical location well (Fig. 1a and Additional file 1: Fig. S1). A subsequent supervised random forest analysis showed that the main environmental driver of these clusters for viral community composition was MAP, MAT, longitude, and latitude. There have been few reports regarding location and climate factors and their effects on the distribution of viruses. Such as the altitude of Antarctic soils which probably linked to temperature could influence microbial metabolism and substantially impact viral communities and functions [16]. The temperature change along the latitude in this study may have similar effects, especially on viral community. All of the viruses differentiating these clusters were unclassified viruses. This highlighted the lack of knowledge and reference sequences for soil viruses.

Although phosphorus is an important factor of viral genome synthesis, the results do not imply any relationship between soil available P content and viral communities and functions. It is possible that our sampling time may be at different stages of phosphorus metabolism because of different fertilization time in each agricultural region. On the other hand, soil available P content may affect viral abundance more than viral community composition, and we will further focus on this point in the future.

### Viruses may directly manipulate P cycling in soils

The *phoH* gene has been widely used as a signature gene for assessing viral phylogeny and diversity, and is encoded by various morphologically distinct viruses that infect a wide range of hosts, including autotrophic and heterotrophic Bacteria and Eukaryotes [30, 57]. A diversity of *phoH* genes have been found in viral communities inhabiting numerous environments, such as seawater [30], paddy water [57], and a Namib hypolith [11]. In these studies, *phoH* genes were distributed according to depth and location [30], biogeography [57], or were found to be entirely novel [11]. In this study, phylogenetic analyses showed that *phoH* sequences in groups 1, 2, and 3 (Fig. 3) were widely distributed in soils [57] from different sites of the world [17, 58, 59]. Group 4 and 5 contained viruses from different environments, including fresh water, sea water, and soil. The majority of the Namib hypolith *phoH* amino acid sequences clustered separately from other sequences and was omitted from our phylogenetic tree. These results support the inference that the distribution of viral *phoH* genes is more dependent on characteristics of the environment [66].

**Fig. 5:**
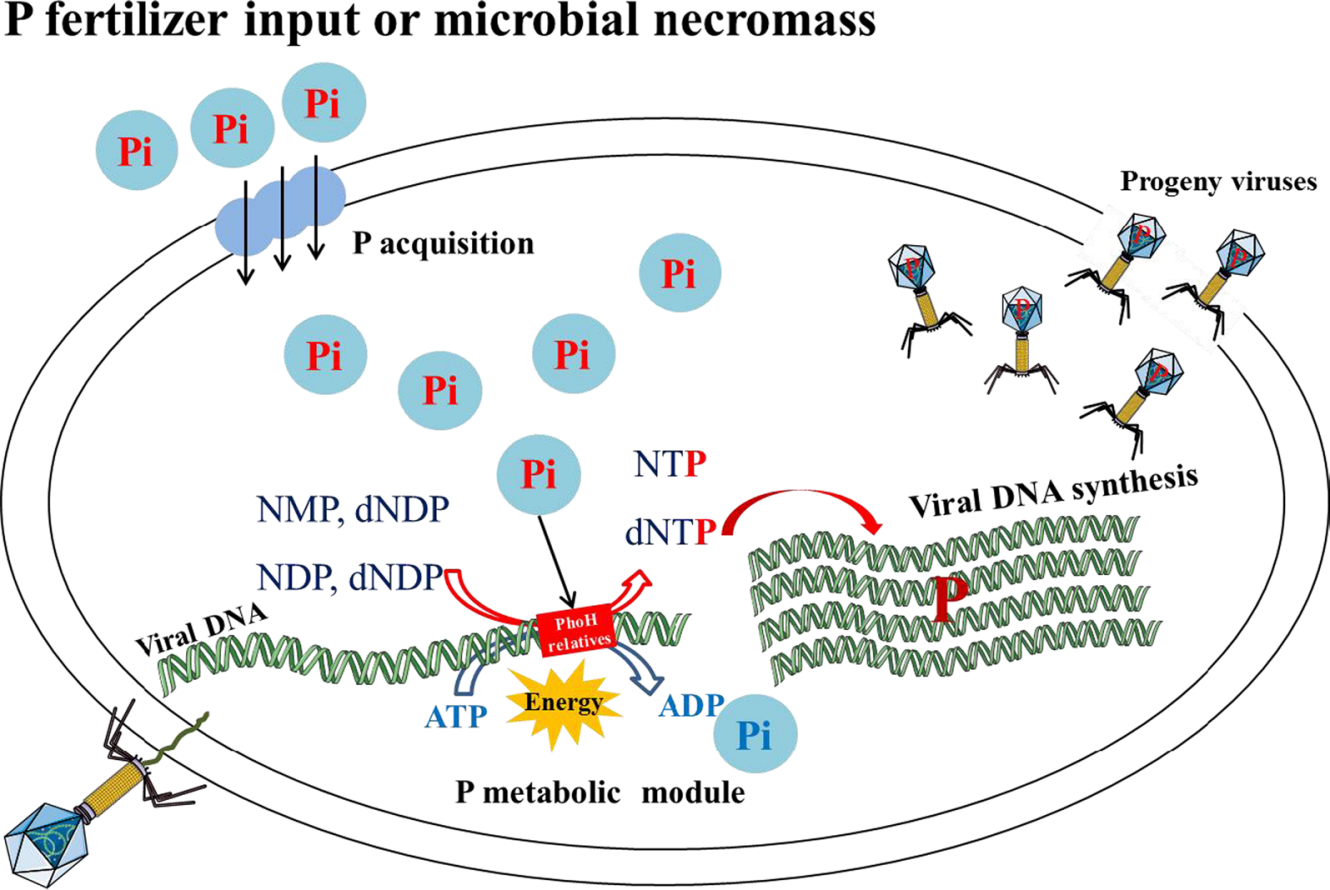
Conceptual model of the P metabolism module of viruses

During the second Chinese soil survey [67], a database created from 2473 soil profiles was analyzed and showed relatively consistent C:P (136) and N:P (9.3) ratios, with a highly constrained C:N:P ratio of 134:9:1 for the surface soils from both of agricultural and natural soils [68]. This ratio indicates that the P content in Chinese soils is generally lower than that required by phages, which have a C:N:P ratio of 20:6:1 [20]. Due to P slow diffusion and high fixation in soils, plus the crops on the absorption of P for agricultural production [19], this means that P can be a major limiting factor for soil microbes, especially viruses. Based on this background, this P deficient environment may select for these viruses to regulate P uptake and metabolism through evolution of the *phoH* gene. It is interesting that all eight *phoH* gene sequences identified in this study were from viruses in agricultural soils. It is possible that agricultural soil is a rich environment in terms of dissolved organic matter, produced via photosynthesis, and nitrogen applied as fertilizer, but that these excesses of C and N result in P being limited. Once P fertilizer input, virus may prompt its host to quickly absorb inorganic P (Pi) and use PhoH to promote its own reproduction (Fig. 5).

To better understand the metabolic potential of *phoH* genes, we searched for, but did not find, additional genes in the Pho regulon. However, it is interesting that four auxiliary metabolic potentials related to nucleotide synthesis, including dUTPase, MazG, Thy1, and RNR, were identified in association with *phoH* to act as a P metabolism module. Previous studies have demonstrated the presence of at least five proteins involved in P metabolism including PhoH, RNR, Thy1, endodeoxyribonuclease, and MazG pyrophosphatase in marine phage genomes [53, 54]. Similar modules were also found in two complete viral genomes from two agricultural soils in our previous data [17], including dUTPase, PhoH, RNR, and Thy1 (Additional file 1: Fig. S3). Here, five of the P metabolism genes were identified, especially in agricultural soils (Fig. 4). Among them, MazG is reported as a nucleoside triphosphate pyrophosphohydrolase, which can hydrolyze all eight of the canonical ribo- and deoxynucleoside triphosphates to their respective monophosphates and PP(i), with a preference for deoxynucleotides [69]. RNR, known as ribonucleoside diphosphate reductase, converts all four ribonucleotide diphosphates (rNDPs) to the respective deoxynucleoside diphosphates (dNDPs), which are then rapidly converted to dNTP [53, 70]. The dUTPase can catalyze dUTP to dUMP and release diphosphate, and provide a substrate (dUMP) for thymidylate synthase [52]. Thy1 can convert dUMP to dTMP depending on FAD, NADPH and 5,10-methylenetetrahydrofolate [71]. PhoH has been reported as a cytoplasmic protein with an ATP-binding activity and is predicted to be induced by P starvation [29]; however, its function remains unknown. Altogether, this information led us to hypothesize that PhoH can act as a nucleotide synthase, possibly binding and hydrolyzing ATP through its conserved nucleoside triphosphate hydrolase domain to obtain energy, and taking advantage of Pi from the agricultural soil (through the host cell) to catalyze the synthesis of nucleotides for the virus's own genome (conceptual model in Fig. 5). This model predicts the proliferation of a huge number of soil viruses playing an important role in depleting P from the soil ecosystem. Future work should focus on whether the concentration of Pi in soil is associated with the number of progeny produced by viruses, and also quantify the contribution of viruses to P loss from soil.

## Conclusions

In summary, our analyses mainly explored viral community structure and function in soils across China. The results revealed that the distribution of viral communities was at least partly determined by geographical location and climate factors. Remarkably, AMGs related to P metabolism, including PhoH, RNR, Thy1, dUTPase and MazG, were mainly identified in viral genomes from agricultural soils, which suggested that viruses possibly take advantage of the Pi added to agricultural soils to synthesize their own genomes. As a consequence, these soil viruses have the potential to significantly contribute to P cycling in the soil ecosystem. Future investigations of the relationship between soil Pi content and viral ecology will reveal the specific mechanism of viral genome synthesis using soil-derived P and resulting depletion of soil P and provide more detailed insights into the contributions of viruses to the P cycle in soil ecosystems.

## Supplementary Information

**Additional file 1.** **Fig. S1.** The distribution of the soil sampling sites. **Fig. S2.** Variable importance plot of contigs from random forest classification analysis based on geographical distribution. **Fig. S3.** Two complete viral genomes contain a P metabolism module.

**Additional file 2.** **Table S1.** Soil sampling information and physicochemical properties. **Table S2.** The amino acid sequences of phoH genes f.rom different environmental samples. **Table S3.** Overview of 19 virome data. **Table S4.** The vOTU table. **Table S5.** The abundance of viral reads identified by Kraken 2 (Family level).

## Data Availability

Virome read data are available in the NCBI Short Read Archive (SRA) under BioProject ID PRJNA579576.

## Abbreviations

P: Phosphorus
VLPs: Virus-like particles
AMGs: Auxiliary metabolic genes
vOTUs: Viral Operational Taxonomic Units
NMDS: Non-metric multi-dimensional scaling
NCBI: National Center for Biotechnology Information
ORFs: Open reading frames
RNR: Ribonucleoside reductase
Thy1: Thymidylate synthase complementing protein
JTT: Jones–Taylor–Thornton
SRA: Short Read Archive
MDA: Multiple displacement amplification
Pi: Inorganic phosphorus
N: North
SE: Southeast
SW: Southwest
EC: Soil electrical conductivity
OM: Soil organic matter
TN: Soil total N content
AP: Available phosphorus
AK: Available potassium
MAP: Mean annual precipitation
MAT: Mean annual temperature
