# Supplementary material for: Distribution of soil viruses across China and their potential role in phosphorous metabolism
Source: Environ Microbiome. 2022 Feb 7;17:6. doi: 10.1186/s40793-022-00401-9 (PMC8822697; doi:10.1186/s40793-022-00401-9)

Fig. S1 The distribution of the soil sampling sites. Red dots represent agricultural soils, and black dots represent natural soils. The samples with initial A were from agricultural soils, and initial N were from natural soils.


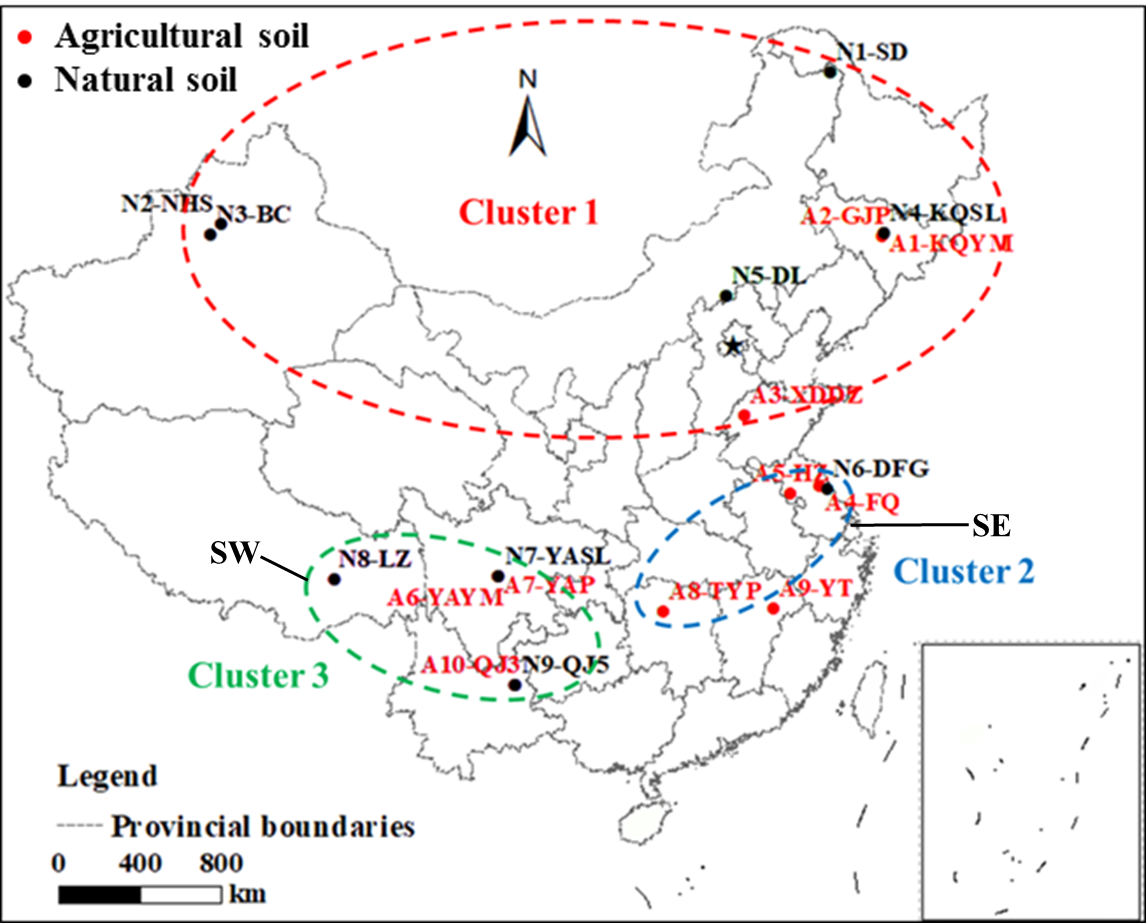


Fig. S2 Variable importance plot of contigs from random forest classification analysis based on geographical distribution. Variables are ranked from highest to lowest according to their mean decrease in accuracy.


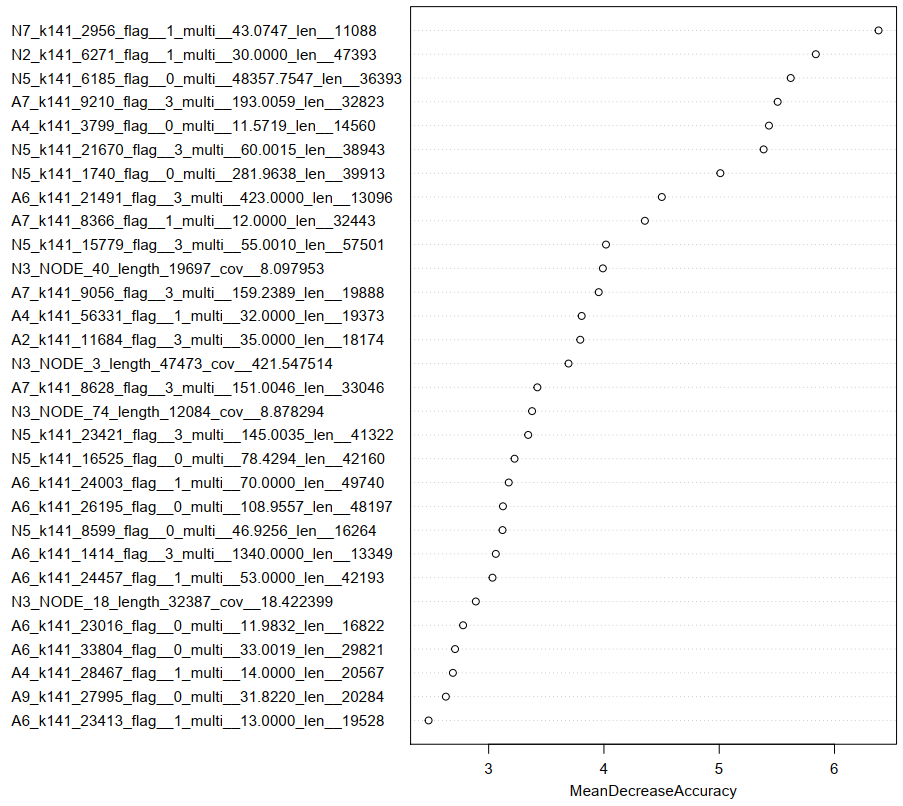


Fig. S3 Two complete viral genomes contain a P metabolism module.


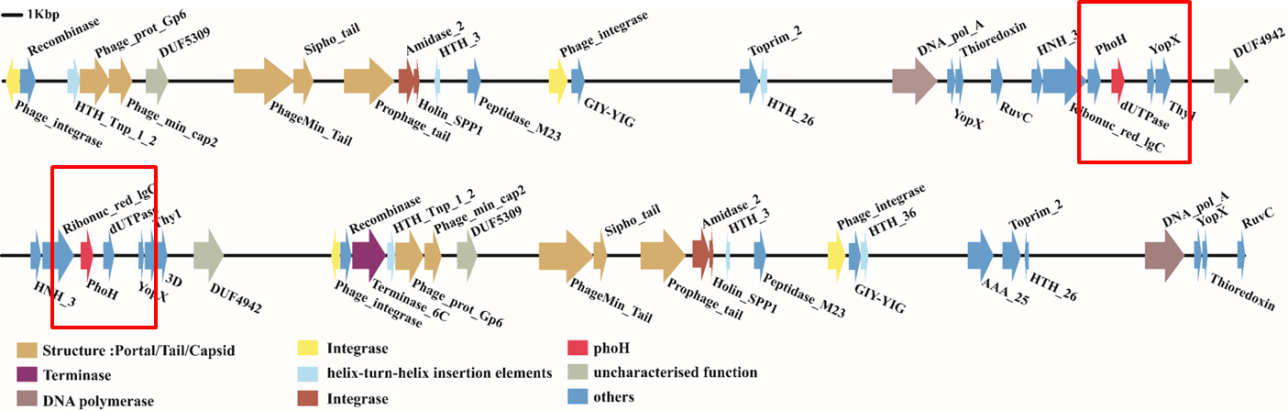

Supplement: Supplementary file 1 — Additional file 1. Fig. S1. The distribution of the soil sampling sites. Fig. S2. Variable importance plot of contigs from random forest classification analysis based on geographical distribution. Fig. S3. Two complete viral genomes contain a P metabolism module. [file 40793_2022_401_MOESM1_ESM.docx]
